# Analysis of Fecal Microbial Changes in Young Calves Following Bovine Rotavirus Infection

**DOI:** 10.3390/vetsci10080496

**Published:** 2023-08-01

**Authors:** Seon-Ho Kim, Youyoung Choi, Michelle A. Miguel, Shin-Ja Lee, Sung-Sill Lee, Sang-Suk Lee

**Affiliations:** 1 Department of Animal Science and Technology, Sunchon National University, Suncheon 57922, Republic of Korea; mamiguel@scnu.ac.kr; 2 Institute of Agriculture and Life Science (IALS), Gyeongsang National University, Jinju 52828, Republic of Korea; dudolboy401@gmail.com (Y.C.); tlswk1000@hanmail.net (S.-J.L.); lss@gnu.ac.kr (S.-S.L.); 3 Division of Applied Life Science (BK21), Gyeongsang National University, Jinju 52828, Republic of Korea

**Keywords:** bovine rotavirus, fecal microbiota, holstein calves, metataxonomic

## Abstract

**Simple Summary:**

We conducted a metataxonomic analysis to compare the taxonomic composition and functional profile of the fecal microbiota in healthy and diarrheic calves across three clinical periods, aiming to gain a better understanding of the changes. The findings here might provide information about pathogenic microbiota in diarrheic calves, which were found to be more enriched than in healthy calves.

**Abstract:**

The objective of the present study was to identify changes in fecal microbiota and predict the functional features of healthy calves and those infected with rotavirus over time. Six Holstein calves (average body weight 43.63 ± 1.19 kg, age-matched within 5–7 d) were randomly selected and distributed into two groups which contained three calves each. Fecal samples were taken 3 days before inoculation and on days 1 and 7 post-inoculation. The 16S rRNA gene amplicon sequencing was performed. Bacterial diversity tended to decrease in the rota group, as indicated by the alpha (evenness, *p =* 0.074 and Shannon, *p =* 0.055) and beta (Bray–Curtis dissimilarity, *p =* 0.099) diversity at 1 day post-inoculation. Differences in the bacterial taxa between healthy and rota-infected calves were detected using a linear discriminant analysis effect size (LDA > 2.0, *p* < 0.05). Rota calves had a higher abundance of certain bacterial taxa, such as *Enterococcus*, *Streptococcus*, and *Escherichia*-*Shigella*, and a lower abundance of bacteria that contribute to the production of short-chain fatty acids, such as *Alistipes*, *Faecalibacterium*, *Pseudoflavonifractor*, *Subdoligranulum*, *Alloprevotella*, *Butyricicoccus*, and *Ruminococcus*, compared to the healthy calves. The observed changes in the fecal microbiota of the rota-infected group compared to the healthy group indicated potential dysbiosis. This was further supported by significant differences in the predicted functional metagenomic profiles of these microbial communities. We suggest that calves infected with bovine rotavirus had bacterial dysbiosis, which was characterized by lower diversity and fewer observed genera than the fecal microbiota of healthy calves.

## 1. Introduction

Calf diarrhea is a common problem in dairy calves and causes significant economic losses to cattle producers. Moreover, calf diarrhea can have significant long-term effects, such as delayed first calving, high rates of morbidity and mortality, growth impairment, and increased treatment costs [1]. Neonatal calf diarrhea has a mortality risk of approximately 5%, making it the leading cause of death among dairy calves within the first month of life [2,3]. This condition is closely linked to the gut microbiota of neonatal calves, which undergo rapid development and experience significant changes in the early stages of life [4]. This rapid development is crucial because calves with neonatal diarrhea exhibit lower bacterial diversity compared to healthy calves. This observation suggests a potential association between host health and gut microbiota, indicating that the composition of the gut microbiota may play a role in calf health [5]. Therefore, many studies have indicated that microbial colonization of the gastrointestinal tract of neonatal calves may commence prior to birth, followed by rapid modifications in the first few weeks of life [6,7].

Improving the development and composition of the gut microbiota is one of the most important strategies for reducing the prevalence of enteric infections. Many studies have highlighted the important role of gut microbiota in animal productivity and health, including the immune system [4]. In addition, several studies have examined alterations in the microbiota and its functional potential during neonatal diarrhea in cattle, with a specific emphasis on the gut microbiota [8]. Through these investigations, researchers have sought to enhance our understanding of how neonatal diarrhea affects the microbiota and its functionality in cattle. There is a reduction in the abundance of butyrate-producing microorganisms and decreased microbial diversity during the first few weeks of life, which contributes to the occurrence of calf diarrhea [9]. The composition and activities of gastrointestinal (GI) microbiota strongly influence the physiological, nutritional, and immunological functions of the gut. Consequently, it is essential to examine the structural and functional roles of the microbiota in diarrheal calves [8]. Researchers have hypothesized that there may be variations in the fecal microbiomes between calves demonstrating systemic clinical symptoms associated with GI disease and those exhibiting diarrhea as the sole symptom [10]. Furthermore, Gomez et al. [11] suggested that host-microbe interactions play a crucial role in regulating various aspects of health. In line with this, investigations have been conducted to examine the relationship between alterations in the fecal microbial community structure of dairy calves and GI disorders [8,12]. Moreover, it has been reported that gut microorganisms are essential for maintaining intestinal homeostasis and that microbiota, or possible changes in the microbial community structure, are associated with disease [12]. Recent studies have reported differences in the fecal microbiota of diarrheal and non-diarrheic calves at earlier stages (within 2 days of birth or between 2 and 13 weeks of development), which is necessary for developing a healthy microbiome [13,14]. However, there is still much to learn about the gut microbiota of both healthy and neonatal calves with diarrhea. In the present study, we hypothesized that infection with rotavirus in Holstein calves leads to alterations in the composition and diversity of the fecal microbiota compared to healthy calves. By conducting sequential sampling at three time points: 3 days before inoculation, 1 day post-inoculation, and 7 days post-inoculation, we can observe the step-by-step impact of rotavirus on the fecal microbiota and gain insights into the dynamics of this infection. Specifically, we expected that rotavirus-infected calves will exhibit decreased bacterial diversity, changes in the relative abundance of specific bacterial taxa, and a shift in functional metagenomic profiles indicative of dysbiosis. Therefore, the present study aimed to analyze the fecal microbial community of neonatal dairy calves in order to investigate potential differences in fecal microbiome profiles between calves with and without diarrhea.

## 2. Materials and Methods

### 2.1. Ethics Statement

All experimental protocols were approved by the Animal Care and Use Committee (approval number: SCNU IACUC-2022-08) of Sunchon National University (Sunchon, Republic of Korea). All the experiments were performed in accordance with the guidelines and regulations set by the governing body. Throughout the study, the health of the calves was closely monitored, including regular assessments of body temperature, body condition score, and any signs of discomfort or illness.

### 2.2. Experimental Design, Animals, and Diet

A cohort of six male Holstein calves from one dairy farm in Jeollanamdo province was used in the study. Holstein calves (average body weight 43.6 ± 1.19 kg, age-matched within 5–7 d) were randomly selected and distributed into two groups, each containing three calves. The healthy (control) group consisted of three healthy calves, whereas the rota group consisted of three calves with diarrhea. The calves in the rota group were orally inoculated with 30 mL of bovine rotavirus cell culture supernatant containing a viral titer of 10^4^ TCID/mL to induce diarrhea. Following the inoculation, the consistency of calf feces was monitored day to day. Fecal consistency was scored using a 4-level scoring scale, as suggested by Renaud et al. [15], with 0 = normal (firm but not hard), 1 = soft (does not hold form, piles but spreads slightly), 2 = runny (spreads readily), and 3 = watery (liquid consistency, splatters). The healthy and rota groups were fed a milk replacer twice daily and provided free access to water, concentrate, and forage. The nutrient contents of the calf milk replacer (Neo Base Co., Ltd., Incheon, Republic of Korea) are provided in Table 1.

Fecal samples were taken 3 days before inoculation (D − 3) and on days 1 (D + 1), and 7 (D + 7) post-inoculation. All fecal samples were collected directly from the rectum using sterile gloves and stored at −80 °C until DNA and RNA extraction. Fecal DNA was extracted using the QIAamp PowerFecal Pro DNA Kit (Qiagen, Hilden, Germany), while RNA extraction was done from fecal suspensions (10% *v*/*v*) by using Ribospin™ vRD II (GeneAll, Seoul, Republic of Korea) following the manufacturer’s instructions.

### 2.3. Real-Time RT-PCR Detection of Bovine Rotavirus

Firstly, in this study, we used the Rapid BoviD-5 Ag Test Kit (Cat. No: RG1302DD) to check for rotavirus, following the test procedure provided by the manufacturer. The presence of Group A rotavirus was detected in fecal samples using qRT-PCR. The primers and probes specific for VP6 were developed by Tsuchiaka et al. [16]. The following primers and probe were: RVA-F: 5′–ACTCCAATGTAAGTGATCTAATTC–3′; RVA-R: 5′–GAGTTGTTCCAAGTAATCCAAA–3′; and RVA-P: 5′–FAM-ACCAATTCCTCCAGTTTGGAAYTCATTYCC-BHQ1–3′. One-step real-time reverse transcription (qRT-PCR) assay was performed in 20 µL total reaction volume containing 2 µL of RNA template, 1 µL of 10 pmol/µL of each primer, 1 µL of probe, 10 µL of Hyperscript One-step 2X RT-PCR Master mix, and 5 µL of nuclease-free water. The thermal cycling condition were 50 °C for 30 min for reverse transcription and 94 °C for 4 min, followed by 40 cycles of denaturation at 94 °C for 30 s, annealing at 55 °C for 1 min, and extension at 72 °C for 1 min, and a final extension at 72 °C for 7 min. Amplification was performed using a CFX96 Touch Real-Time PCR Detection System (Bio-Rad, Hercules, CA, USA). For standard curve calibration, the concentration of the bovine rotavirus standard was as 61.8 ng/μL. An 8-point 10-fold serial standard curve was prepared. Water was used as the non-template control. qRT-PCR amplification was performed using an optimized protocol on a CFX96 Touch Real-Time PCR Detection System (Bio-Rad). For bovine rotavirus detection by qRT-PCR, a Ct value < 35 was considered positive; otherwise, it was considered negative.

### 2.4. Metagenome Analysis

To assess the quality and quantity of the extracted DNA, a NanoDrop ND-2000 spectrophotometer (Thermo Fisher Scientific, Waltham, MA, USA) was utilized. Amplicons were generated to target the V3-V4 region of the 16S rRNA gene and subsequently subjected to sequencing at Macrogen (Seoul, Republic of Korea). Briefly, the 16S rRNA gene amplicon libraries encompassing both bacteria and archaea were amplified using universal primers 341F (5′-CCTACGGGNGGCWGCAG-3′) and 805R (5′-GACTACHVGGGTATCTAATCC-3′) as described by Herlemann et al. [17]. Each DNA sample was assigned a unique barcode. The obtained amplicons were sequenced utilizing the Illumina MiSeq platform in San Diego, CA, USA. For sequence data analysis, QIIME2 (version 2021.11, [18]) was employed. Barcodes and primer sequences were removed using Cutadapt [19]. The forward and reverse reads were denoised and quality-filtered (Q-score > 25) using the DADA2 plugin, followed by merging and removal of chimeras [20]. Afterward, the denoised sequences underwent clustering into amplicon sequence variants (ASVs) through the utilization of a manually trained naïve Bayes taxonomy classifier. The classifier received training based on the Silva (SSU138) 16S rRNA gene database, which had been clustered at 99% similarity specifically for the 341F/805R region [21]. Before downstream analysis, we excluded ASVs identified as unassigned, mitochondria, or chloroplasts. To minimize sampling heterogeneity, the ASV table was rarefied 1000 times per sample to an equal number of reads using the ‘q2-repeat-rarefy’ plugin in QIIME2 [22]. The rarefied ASV table was used to assess microbial diversity, both within samples (alpha diversity) and between samples (beta diversity). Alpha diversity was assessed using Chao1 richness, evenness, Simpson’s index, and Shannon’s index, while beta diversity was analyzed using Bray-Curtis and Weighted UniFrac distances. To predict the metabolic functions (KEGG pathways and modules) derived from the fecal microbiota, we employed the PICRUSt2 tool (v.2.4.1) [23]. The raw 16S rRNA gene amplicon sequencing were submitted to NCBI Sequence Read Archive (accession number: PRJNA931806).

### 2.5. Statistical Analysis

To assess the normality of the data, we conducted Shapiro–Wilk test using SAS 9.4 (SAS Institute Inc., Cary, NC, USA). Normally distributed data were analyzed using Student’s *t*-test. Abnormally distributed data were further analyzed using the non-parametric Wilcoxon rank-sum test. Principal coordinate analysis (PCoA) utilized the obtained distance matrices as inputs, while the significance of sample clustering was assessed through permutational multivariate analysis of variance (PERMANOVA) employing 9999 permutations. For the analysis of differential relative abundances in the fecal microbiota and its predicted metabolic categories, we employed linear discriminant analysis effect size (LEfSe). Significance was determined based on LDA scores exceeding 2.0 and a *p* value threshold of less than 0.05 [24]. For all statistical analyses, significance was set to *p* < 0.05, and a tendency of difference was declared at 0.05 < *p* ≤ 0.10.

## 3. Results

### 3.1. Clinical Findings of Rotavirus in Calves

As shown in Table S1, we checked for clinical signs of bovine rotavirus using qRT-PCR in healthy and rota groups. The Ct value for the healthy group was >35, indicating a negative result. In contrast, the rota group had a lower Ct value, confirming the presence of rotavirus. Table S2 summarizes the fecal consistency scores in the healthy and rota groups. Following the inoculation with bovine rotavirus, the rotavirus group exhibited higher scores than the healthy group.

### 3.2. Quality Evaluation and Sample Statistics

We performed 16S rRNA gene amplicon sequencing of fecal samples collected per rectum at three time points: 3 days before inoculation, one day post-inoculation, and 7 days post-inoculation, to determine the temporal changes in the calf gut microbiota in response to the rota treatment. A total of 2,157,400 amplicon sequences were generated from fecal samples from the healthy and rotavirus-infected groups. After applying a quality filter (Q score > 25) using QIIME2, we obtained 1,161,065 sequences, with an average of 64,503 ± 14,358 sequences per sample. For all samples, the calculated Good’s coverage was above 99%. In this study, the detected bacterial taxa were defined as those with a relative abundance above 0.1% and a presence in more than 50% of the animals per group.

### 3.3. Diversity Analysis of Fecal Microbiota in Healthy and Rota Groups

**Figure 1:**
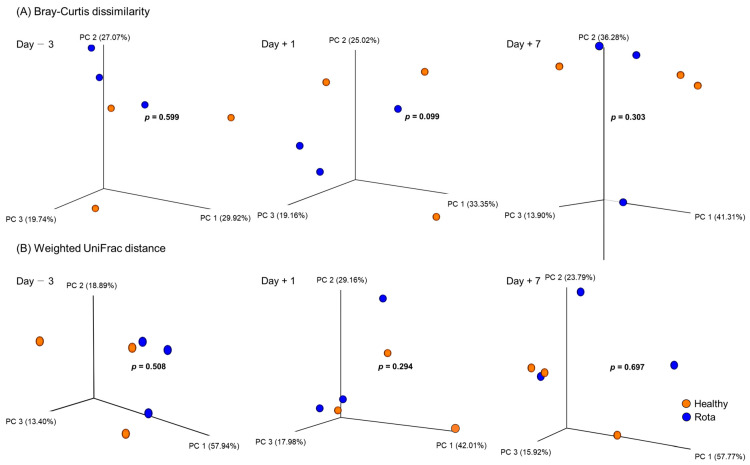
Change in the fecal bacterial community structure in Holstein calves. PCoA plot was generated based on (**A**) Bray–Curtis dissimilarity and (**B**) Weighted UniFrac distance in fecal bacterial communities determined via 16S rRNA gene amplicon sequencing for respective ages; 3 days before inoculation, 1 day post-inoculation, and 7 days post-inoculation. Individual points in each plot represent individual animals. Colors indicate the groups; healthy (orange) and rota (blue). *p* values were calculated using PERMANOVA test (*n* = 9999).

Alpha diversity measurements of the fecal microbiota of the healthy and rotavirus-infected groups are shown in Table 2. On 1 day post-inoculation with bovine rotavirus, evenness (*p =* 0.074) and Shannon’s index (*p =* 0.055) tended to be higher in the healthy group than in the rotavirus group, whereas other measurements did differ. There was no difference in the alpha diversity measurements between the healthy and rota groups on D − 3 and D + 7. With regard to beta diversity analyzed using PERMANOVA, the Bray–Curtis dissimilarity in the rota group (*p =* 0.099) tended to differ from that healthy group on D + 1, whereas no differences was observed for D − 3 and D + 7 (Figure 1). Moreover, the weighted UniFrac distance did not show any differences in the fecal microbiota of the healthy and rotavirus-infected groups at any sampling period.

### 3.4. Taxonomic Composition of Fecal Microbiota in Healthy and Rota Groups

**Figure 2:**
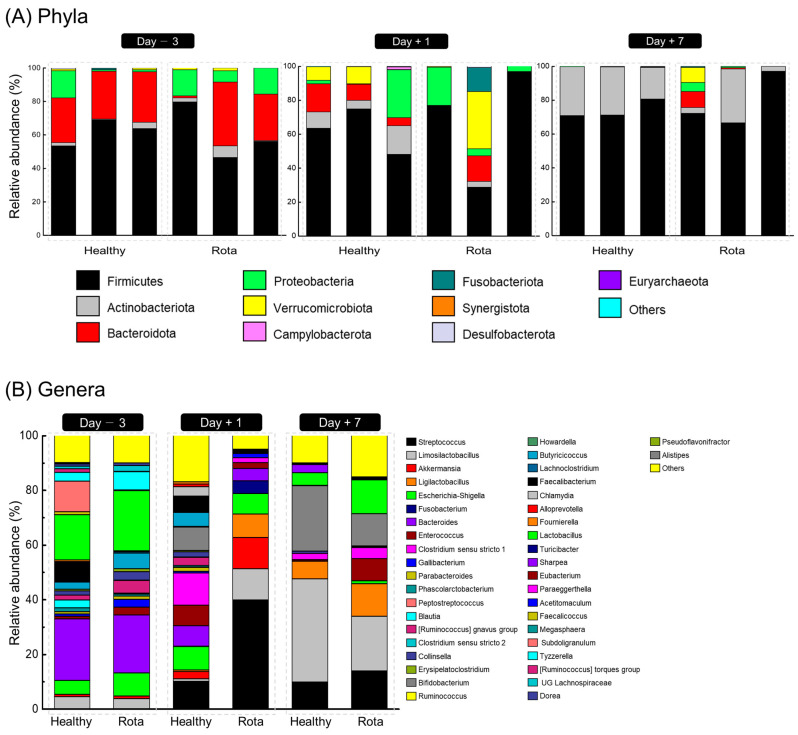
Fecal bacterial compositional profiles of Holstein calves. (**A**) Relative abundance of major bacteria phyla (relative abundance ≥ 0.1% in more than 50% animals) for all individuals. (**B**) Relative abundance of major bacteria genera (relative abundance ≥ 0.1% in more than 50% animals) for all individuals.

The taxonomic distributions of the fecal microbiota at the phylum and genus levels are shown in Figure 2. Three days before oral challenge with bovine rotavirus, 7 phyla, 17 families, and 26 genera were identified (Table S3). No significant differences were observed between the healthy and rotavirus groups at the phylum level. However, two families and four genera differed between the healthy and rotavirus groups at the family and genus levels. At the family level, the relative abundance of Ruminococcaceae (18.90% vs. 0.55% for healthy and rota, respectively; *p =* 0.081), Oscillospiraceae (0.27% vs. 0.01% for healthy and rota, respectively; *p =* 0.077), and Rikenellaceae (0.16% vs. 0.00% for healthy and rota, respectively; *p =* 0.077) was higher in the healthy group than in the rota group. At the genus level, the relative abundance of *Faecalibacterium* (7.24% vs. 0.39% for healthy and rota, respectively; *p =* 0.081), *Subdoligranulum* (11.10% vs. 0.11% for healthy and rota, respectively; *p =* 0.077), *Pseudoflavonifractor* (0.27% vs. 0.01% for healthy and rota, respectively; *p =* 0.077), and *Alistipes* (0.16% vs. 0.00% for healthy and rota, respectively; *p =* 0.077) tended to be higher in the healthy group than rota group.

On day 1 post-inoculation with bovine rotavirus, 9 phyla, 20 families, and 25 genera were identified (Table S4). At the phylum level, Actinobacteriota (10.50% vs. 1.23% for healthy vs. rotavirus groups, respectively; *p =* 0.064) tended to be more abundant in the healthy than rota group. At the family and genus levels, three families and two genera differed between the healthy and rotavirus groups. Briefly, at the family level, the relative abundance of Butyricicoccaceae (5.07% vs. 0.00% for healthy and rota, respectively; *p =* 0.077) and Prevotellaceae (0.98% vs. 0.00% for healthy and rota, respectively; *p =* 0.064) was higher in the healthy group than in the rota group. At the genus level, the relative abundance of *Butyricicoccus* (5.07% vs. 0.00% for healthy and rota, respectively; *p =* 0.077) and *Alloprevotella* (0.98% vs. 0.00% for healthy and rota, respectively; *p =* 0.064) was higher in the healthy group than in the rota group.

On day 7 post-inoculation with bovine rotavirus, 7 phyla, 13 families, and 16 genera were identified (Table S5). At the phylum level, no significant differences were observed between the healthy and rotavirus-infected groups. However, at the family and genus levels, one family and one genus differed between the healthy and rotavirus groups. At the family and genus levels, Enterococcaceae (0.43% vs. 8.16% for healthy and rotavirus groups, respectively; *p =* 0.081) and *Enterococcus* (0.43% vs. 8.16% for healthy and rotavirus groups, respectively; *p =* 0.081) were higher in the rotavirus group than in the healthy group.

**Figure 3:**
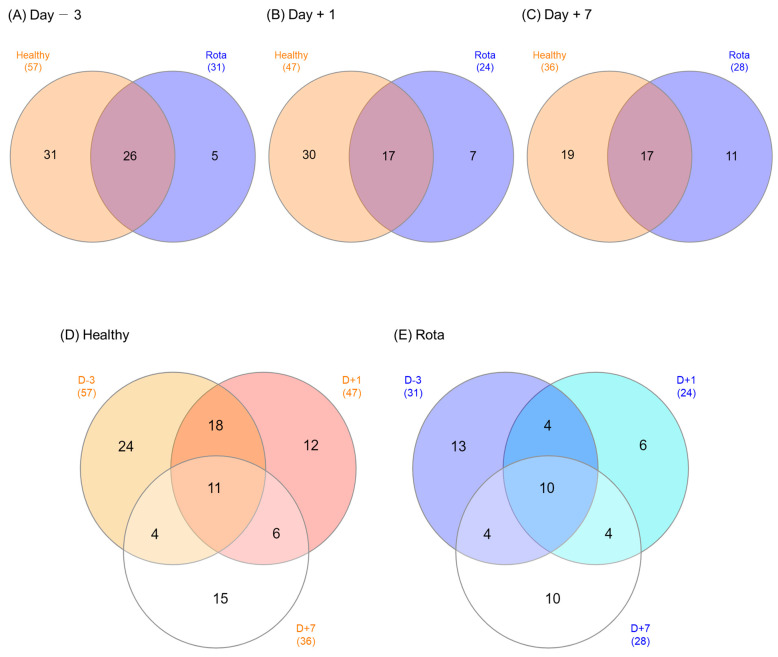
Venn diagrams showing the genera of fecal microbiota shared between the healthy and rota groups. (**A**) 3 days before inoculation, (**B**) 1 day post-inoculation, (**C**) 7 days post-inoculation, (**D**) Healthy group, and (**E**) Rota group.

Venn diagrams were used to illustrate the microbial genera shared by, or exclusively detected in, the healthy and rotavirus groups (Figure 3). On D − 3, 26 of the 62 detected genera were shared between the healthy and rotavirus-infected groups (31 vs. 6, respectively). Of the 54 genera detected on D + 1, 17 were shared, whereas 30 and 7 were exclusively found in the healthy and rotavirus groups, respectively. Similarly, on D + 7, 17 of the 47 detected genera were shared between the healthy and rotavirus groups (19 vs. 11, respectively). The total number of shared microbial genera across all three days was 11 in the healthy group and 10 in the rotavirus group.

**Figure 4:**
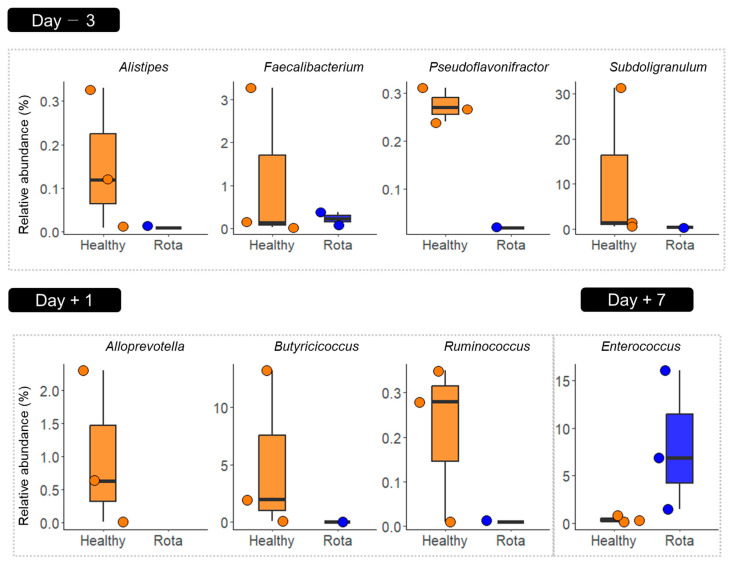
Differentially abundant fecal microbial taxa between healthy and rota groups detected using LEfSe with an LDA effect size > 2. Only classified prevalent taxa (each detected in at least 50% of the samples) were visualized. Data points represent individual animals. LDA: linear discriminant analysis; LEfSe: LDA effect size.

We identified potential taxonomic biomarkers in the fecal microbiota of the calves using LEfSe analysis (Figure 4). Compared to the rota group, the healthy group was differentially enriched at the genus level. The enriched genera included *Alistipes*, *Faecalibacterium*, *Pseudoflavonifractor*, and *Subdoligranulum* on D − 3 and *Alloprevotella*, *Butyricicoccus*, and *Ruminococcus* on D + 1. In contrast, *Enterococcus* was enriched in the rota group compared to the healthy group on D + 7.

### 3.5. Predicted Kyoto Encyclopedia of Genes and Functional Pathways in the Fecal Microbiota in Healthy and Rota Groups

**Figure 5:**
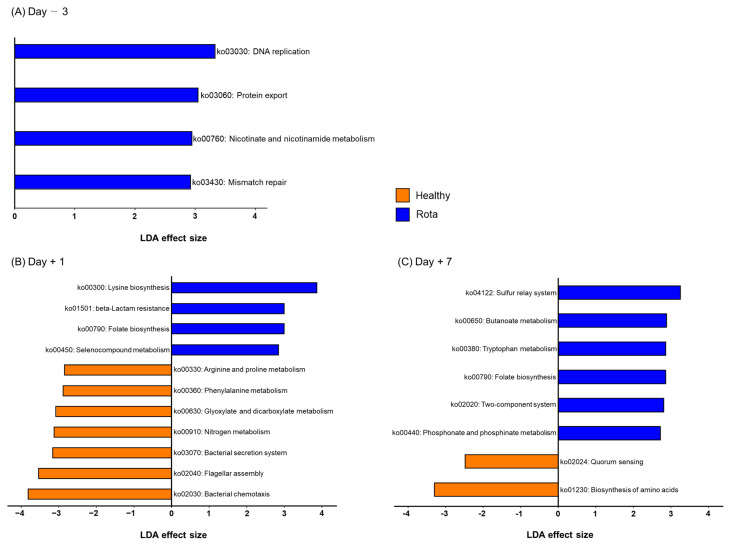
Differentially abundant KEGG pathways between healthy and rota groups detected using LEfSe with an LDA effect size > 2. Only classified prevalent taxa (each detected in at least 50% of the samples) were visualized. KEGG: Kyoto Encyclopedia of Genes and Genomes; LDA: linear discriminant analysis; LEfSe: LDA effect size.

To predict functional biomarkers in the fecal microbiota of the healthy and rotavirus-infected groups, we performed PICRUSt2 analyses (Table S6). KEGG orthologs (D − 3: 4987 vs. 4647; D + 1: 4897 vs. 5065; and D + 7: 4633 vs. 4371) were predicted in the healthy and rotavirus groups, respectively. In addition, KEGG pathways (D − 3: 127 vs. 126; D + 1: 136 vs. 139; and D + 7: 246 vs. 234) and KEGG modules (D − 3: 243 vs. 250; D + 1: 140 vs. 126; and D + 7: 232 vs. 228) were predicted in the healthy and rota groups, respectively. Based on LEfSe analysis, we identified 23 of the predicted KEGG pathways that were significantly altered in the healthy and rotavirus groups on D − 3, D + 1, and D + 7 (Figure 5). All KEGG pathways (ko03030: DNA replication; ko03060: Protein export; ko00760: Nicotinate and nicotinamide metabolism; and ko03430: Mismatch repair) were enriched in the rota group compared with the healthy group on D − 3. However, on D + 1, some KEGG pathways were differentially enriched between the healthy and rotavirus-infected groups. Briefly, seven KEGG pathways, including ko00330: Arginine and proline metabolism; ko00360: Phenylalanine metabolism; ko00630: Glyoxylate and dicarboxylate metabolism; ko00910: Nitrogen metabolism; ko03070: Bacterial secretion system; ko02040: Flagellar assembly; and ko02030: Bacterial chemotaxis were enriched in the healthy group. In constrast, other KEGG pathways, including ko00300: Lysine biosynthesis; ko01501: beta-lactam resistance; ko00790: Folate biosynthesis; and ko00450: Selenocompound metabolism were enriched in rota group. Furthermore, on D + 7, KEGG pathways ko02024: Quorum sensing and ko01230: Biosynthesis of amino acids were enriched in the healthy group, whereas KEGG pathways, including ko04122: Sulfur relay system; ko00650: Butanoate metabolism; ko00380: Tryptophan metabolism; ko00790: Folate biosynthesis; ko02020: Two-component system; and ko00440: Phosphate and phosphinate metabolism were enriched in rota group. We also found that 39 of the predicted KEGG modules (D − 3: 3 and 2; D + 1: 10 and 12; D + 7: 5 and 7; healthy and rota groups, respectively) were significantly altered in the healthy and rota groups (Table S7).

## 4. Discussion

The present study was designed to investigate changes in the fecal microbiota of Holstein calves, following rotavirus challenge, while minimizing the effects of external factors. Male calves were selected for each group based on age and body weight to ensure high nutritional and physiological homogeneity. The study participants were maintained under the same living conditions and fed the same diet throughout the experimental period, with no significant alterations in their diet. The primary source of nutrition for calves during the study was milk replacement. These measures were taken to control for potential confounding variables and to enable a more accurate investigation of the effects of other factors on the fecal microbiota of calves. Despite controlling for external factors, subtle differences in gut microbiota composition may have existed at baseline, which was not detected by the study design or fecal microbiota analysis. Additionally, the rotavirus challenge may have indirectly affected the gut microbiota prior to D + 1 infection through an immune response or other mechanisms.

On day 1 post-inoculation with bovine rotavirus, we found that bacterial diversity decreased in the rota group via alpha (evenness and Shannon’s index) and beta (Bray–Curtis dissimilarity) diversity, which are well-known characteristics of dysbiosis [1,25]. This result is consistent with that of a previous study that analyzed the fecal microbiota of Holstein diarrheic calves [25,26]. These findings suggest that the rota group had significant dysbiosis in the GI tract, with lower bacterial diversity and fewer bacterial genera than the healthy group, indicating marked differences in their overall microbial structure. Additionally, the incidence of diarrhea in calves during the early stages of life is associated with low bacterial diversity in the gut [9]. However, another previous study reported no significant difference in alpha diversity measurements between healthy and diarrheic groups, although both groups of bacterial communities were clearly discriminated [27]. Interestingly, we found no significant difference in alpha diversity between the healthy and rota groups, in contrast to the previously mentioned study. This suggests that while rotavirus may not directly alter alpha diversity in all cases, it can still impact the bacterial community. It is worth noting that the number of days after rotavirus infection can have distinct effects on the microbial communities of dairy calves, influencing alpha diversity in different ways. This highlights the importance of considering the specific stage of infection when assessing the impact on fecal microbial communities.

Most of the bacterial genera were enriched in the healthy group compared to the rotavirus group. The most important commensal bacterial genera in the healthy group were *Alistipes*, *Faecalbacterium*, *Pseudoflavonifractor*, *Subdoligranulum*, *Alloprevotella*, *Butyricicoccus*, and *Ruminococcus*; most of these were also found in previous studies [9,27]. *Alistipes* and *Alloprevotella*, which belong to the *Bacteroidetes*, contribute to the production of short-chain fatty acids (SCFA) and further benefit gut health [28]. *Faecalbacterium* and *Pseudoflavonifractor* are beneficial for the immune homeostasis of calves, and are positively correlated with bovine IgG [27]. In addition, a high abundance of *Faecalibacterium* is associated with less diarrhea in neonatal calves, and this genus can produce butyrate in the gut [29]. Butyrate-producing genera, such as *Subdoligranulum*, *Butyrcicocus*, and *Ruminococcus*, were also enriched in the healthy group, which may act as probiotic features that positively influence the gut health of young calves, as suggested by Xin et al. [30]. In particular, *Butyricicoccus* can strengthen the epithelial barrier function or defend the gut from pathogens in neonatal calves [14]. We speculate that the high prevalence of SCFA-enhancing genera in the healthy group may be associated with the robustness of their gut health. Short chain fatty acids are important for maintaining host gut health, as they serve as a major source of energy for the colonic mucosa. Among them, butyrate is not only a nutrient source for gut colonocytes but is also thought to be beneficial for the immunological maturation of the gut mucosa [31]. Although not identified as a potential taxonomic biomarker by LEfSe analysis, *Bifidobacterium* was high, and its relative abundance in the healthy group, by more than 8% (8.53% vs. 0.03%) on D + 1. This genus has primary health-promoting functions in calves, with preventive and protective effects against diarrhea and intestinal infections [32]. The high relative abundance of this genus in the healthy group benefit gut health. In contrast, the enrichment of *Enterococcus* in the Day + 7 rota group suggests that the composition of the fecal microbiome may have been affected by rotavirus infection over time, emphasizing the potential long-term impact of rotavirus infection on the microbiome. It is possible that the immune response to rotavirus infection or other indirect effects of the infection could have altered the fecal microbiota, leading to differences in *Enterococcus* abundance [33]. These findings highlight the potential long-term effects of rotavirus infection on the fecal microbiota and the need to further investigate the mechanisms underlying these effects. Additionally, the higher abundance of *Streptococcus* and *Escherichia-Shigella* in the rota group, which have been reported as opportunistic pathogens that can cause infections in immunocompromised hosts, may indicate an increased risk of gut infections and inflammation in calves [14,34,35]. Furthermore, rotavirus-infected calves had significantly reduced bovine IgG levels, suggesting that the immune response to rotavirus may have compromised the calves’ immune systems, potentially contributing to the observed differences in microbial composition [36]. These findings suggested that the healthy group had a higher predominance of genera that play important roles in maintaining gut health by modulating the gut immune system, whereas the rotavirus group did not. Future studies should investigate whether other pathogens or infections have similar effects on the gut microbiota and whether the observed differences in *Enterococcus* and *Streptococcus* abundance have any clinical implications for the health of Holstein calves.

Interestingly, we found that some of the predicted metabolic functions of the fecal microbiota were altered between the healthy and rota groups. In the healthy group, bacterial chemotaxis and secretion systems, quorum sensing, and amino acid biosynthesis were enriched. Bacterial chemotaxis and secretion systems are known as bacterial colonization and proliferation pathways, which are required for substrate transport and utilization and are ultimately crucial for energy extraction and provision [37]. Bacteria communicate with each other via a process known as quorum sensing. This mechanism involves using hormone-like signals to mediate commensal and pathogenic interactions between gut microbes and their hosts [38]. Quorum sensing enables bacteria to detect changes in the levels of host and microbial molecules and adjust their functions accordingly by increasing, decreasing, or suppressing the production of metabolites [39]. Thus, we speculate that the high prevalence of SCFA-producing genera in the healthy group may be associated with the upregulation of these functions. In contrast, folate biosynthesis, beta-lactam resistance, the two-component system, and amino acid metabolism (including lysine biosynthesis, selenocompound metabolism, phosphonate and phosphinate metabolism, and tryptophan metabolism) were enriched in the rota group. The gut microbiota of babies, particularly those dominated by *Bifidobacterium*, are enriched in genes involved in folate biosynthesis, which has been verified in previous studies [40,41]. However, in the present study, no significant difference was observed between the healthy and rotavirus-infected groups regarding the presence of *Bifidobacterium*. Nonetheless, folate may also play a role in promoting immune function and metabolism [42]. It is also involved in the base synthesis and is critical in protein synthesis and cell division [43]. Notably, the upregulation of genes responsible for amino acid metabolism in the rota group suggests an imbalance in protein nutrient availability in calves with diarrhea. This finding is consistent with previous research, indicating that improper amino acid metabolism is a significant factor associated with diarrhea [44]. Thus, our results highlight the importance of monitoring and managing protein intake and metabolism in calves with diarrhea. The two-component system function was enriched in the rotavirus group. This function is primarily associated with the *Escherichia* genus, which can produce a barrier effect against enteropathogens [45]. However, if this beneficial metabolism is weakened, it may contribute to the development of diarrhea [27]. Nevertheless, it is important to note that the PICRUSt2 analysis used in this study could only predict metagenomic function, and the use of additional omics tools, such as metabolomics, are recommended to investigate the actual changes in the metabolic function of the microbiota of the rotavirus group further. These findings underscore the need for continued research on the gut microbiota of Holstein calves and the development of strategies to manage and promote their health.

However, it is important to note that the present study used a limited number of animals, and as such, its results should be interpreted with caution. Despite the limited number of calves used in this study, our research provides valuable insights into the temporal changes in the fecal microbiota of Holstein calves following rotavirus infection. Sequential sampling at 3 days before inoculation, 1 day post-inoculation, and 7 days post-inoculation allowed us to observe the step-by-step impact of rotavirus on the fecal microbiota and gain insights into the dynamics of this infection.

## 5. Conclusions

In the present study, we observed differences in fecal microbiota composition and predicted functional metagenome profiles between healthy and rotavirus-infected calves at three stages: 3 days before inoculation, 1 day post-inoculation, and 7 days post-inoculation. We found that the rota group had a higher abundance of certain bacterial taxa, such as *Enterococcus*, *Streptococcus*, and *Escherichia-Shigella*, and a lower abundance of bacteria that contributed to the production of SCFA, such as *Alistipes*, *Faecalibacterium*, *Pseudoflavonifractor*, *Subdoligranulum*, *Alloprevotella*, *Butyricicoccus*, and *Ruminococcus* than the healthy group. Moreover, functional analysis revealed differences in metabolic pathways between the two groups, such as the enrichment of pathways related to amino acids, cofactors, and vitamin metabolism in the rotavirus group. These findings suggest that bovine rotavirus can alter the fecal microbiota composition and functional potential in calves, potentially impacting their overall health and well-being. A major goal of the dairy production system is to optimize diarrhea prevention without relying on antibiotics. Therefore, our results, which demonstrate that rotavirus infection alters the structure of the fecal microbiota and is linked to changes in metabolic functions, not only provide new insight into the treatment of rotavirus-mediated diarrhea in calves but may also be beneficial to the dairy production.

## Figures and Tables

**Table 1:** Chemical composition (as-fed basis) of milk replacer.

Item	Diet
Crude protein	20.0% or more
Crude fat	10.0% or more
Calcium	0.7% or more
Phosphorus	1.5% or less
Crude fiber	3.0% or less
Crude Ash	12.0% or less
Vitamin A	More than 25,000 IU/kg

Values are concentrations declared by the manufacturer.

**Table 2:** Summary of alpha diversity measurements of the fecal microbiota for the healthy and rota groups.

Measurements	Group		SEM	*p* Value
	Healthy	Rota		
Day − 3				
Chao1 estimate	160	79.0	28.5	0.247
Evenness	0.62	0.60	0.04	0.800
Shannon’s index	4.42	3.76	0.25	0.147
Simpson’s index	0.89	0.87	0.03	0.713
Day + 1				
Chao1 estimate	136	87.0	24.2	0.234
Evenness	0.63	0.48	0.04	0.074
Shannon’s index	4.43	3.03	0.35	0.055
Simpson’s index	0.92	0.77	0.04	0.139
Day + 7				
Chao1 estimate	105	99.3	47.7	0.937
Evenness	0.55	0.59	0.03	0.447
Shannon’s index	3.56	3.68	0.62	0.895
Simpson’s index	0.85	0.87	0.04	0.795

Day − 3, 3 days before rotavirus inoculation; Day + 1, 1 day post-inoculation; Day + 7, 7 days post-inoculation. Healthy: non-diarrhea. Rota: calves inoculated with rotavirus to induce diarrhea. SEM: standard error of the mean.

## Data Availability

All data generated or analyzed during this study are available from the corresponding author upon reasonable request.

## Supplementary Materials

The following supporting information can be downloaded at: https://www.mdpi.com/article/10.3390/vetsci10080496/s1, Table S1: qRT-PCR results for bovine rotavirus detection in individual calve feces, Table S2: Individual calves fecal consistency scores in Healthy and Rota groups, Table S3: Relative abundance (≥0.1%) of fecal bacterial phyla, families, and genera between Healthy and Rota groups three days before inoculation, Table S4: Relative abundance (≥0.1%) of fecal bacterial phyla, families, and genera between Healthy and Rota groups post-inoculation day 1, Table S5: Relative abundance (≥0.1%) of fecal bacterial phyla, families, and genera between Healthy and Rota groups post-inoculation day 7, Table S6: Predicted KEGG hierarchies (orthologs, modules, and pathways) between Healthy and Rota groups, Table S7: Differentially abundant KEGG modules between Healthy and Rota groups, which were detected using LEfSe with an LDA effect size > 2.
