# Supplementary material for: Analysis of Fecal Microbial Changes in Young Calves Following Bovine Rotavirus Infection
Source: Vet Sci. 2023 Aug 1;10(8):496. doi: 10.3390/vetsci10080496 (PMC10459456; doi:10.3390/vetsci10080496)
Supplement: Supplementary file 1 [file vetsci-10-00496-s001.zip › vetsci-2485354-supplementary.pdf]

Table S1 qRT-PCR results for bovine rotavirus detection in individual calve feces

Animal no.	Day – 3		Day + 1		Day + 7	
	[1]Ct value	P/N	Ct value	P/N	Ct value	P/N
Healthy 1	> 35	-	> 35	-	> 35	-
Healthy 2	> 35	-	> 35	-	> 35	-
Healthy 3	> 35	-	> 35	-	> 35	-
Rota 1	> 35	-	26.9	+	29.3	+
Rota 2	> 35	-	32.2	+	34.9	+
Rota 3	> 35	-	24.9	+	25.1	+

Day − 3, 3 days before rotavirus inoculation; Day + 1, 1 day post-inoculation; Day + 7, 7 days post-inoculation.
Healthy: non-diarrhea.
Rota: calves inoculated with rotavirus to induce diarrhea.
[1]Ct value >35 is negative.

Table S2 Individual calves fecal consistency scores in Healthy and Rota groups

Animal no.	Day – 3	Day + 1	Day + 7
Healthy 1	0	1	1
Healthy 2	1	1	1
Healthy 3	0	1	1
Rota 1	1	2	2
Rota 2	1	2	3
Rota 3	1	3	3

Day − 3, 3 days before rotavirus inoculation; Day + 1, 1 day post-inoculation; Day + 7, 7 days post-inoculation.
Healthy: non-diarrhea.
Rota: calves inoculated with rotavirus to induce diarrhea.

Table S3. Relative abundance (≥ 0.1%) of fecal bacterial phyla, families, and genera between Healthy and Rota groups three days before inoculation

Measurements	Relative abundance (%)		SEM	*p* Value
	Healthy	Rota		
Day – 3 Phyla				
Firmicutes	62.1	60.8	7.21	0.906
Bacteroidota	28.5	22.3	6.04	1.000
Proteobacteria	6.05	12.7	4.04	0.663
Actinobacteriota	2.02	3.27	1.43	0.591
Verrucomicrobiota	0.73	0.92	0.42	0.663
Fusobacteriota	0.34	0.01	0.17	1.000
Desulfobacterota	0.19	0.01	0.09	0.354
Day – 3 Families				
Lactobacillaceae	21.1	25.9	11.5	0.789
Bacteroidaceae	22.6	21.2	7.71	0.909
Lachnospiraceae	11.3	15.2	4.94	0.641
Enterobacteriaceae	5.13	8.48	3.99	0.586
Butyricicoccaceae	2.67	5.72	1.86	0.328
Coriobacteriaceae	1.51	3.05	1.48	0.516
Pasteurellaceae	0.81	2.92	1.84	0.663
Enterococcaceae	0.93	2.89	1.45	0.420
Tannerellaceae	0.97	1.17	0.80	0.878
Erysipelatoclostridiaceae	0.60	1.11	0.45	0.470
Akkermansiaceae	0.72	0.92	0.42	0.756
Acidaminococcaceae	1.36	0.76	0.51	0.465
Ruminococcaceae	18.9	0.55	7.88	**0.081**
Erysipelotrichaceae	1.14	0.15	0.59	0.663
Clostridiaceae	0.01	0.12	0.04	0.354
Oscillospiraceae	0.27	0.01	0.01	**0.077**
Rikenellaceae	0.16	0.00	0.05	**0.077**
Others	9.85	9.96	6.57	1.000
Day – 3 Genera				
Lactobacillus	16.5	22.0	10.5	0.731
Bacteroides	22.6	21.2	7.71	0.909
Escherichia-Shigella	5.13	8.48	3.99	0.586
Tyzzerella	3.22	6.62	2.95	0.463
Butyricicoccus	2.67	5.72	1.86	0.328
[Ruminococcus] gnavus group	1.84	4.69	2.20	0.454
Limosilactobacillus	4.62	3.85	2.18	0.819
Collinsella	1.51	3.05	1.48	0.516
Gallibacterium	0.81	2.92	1.84	0.663

Enterococcus	0.93	2.89	1.45	0.420
UG Lachnospiraceae	0.90	2.23	1.43	1.000
Parabacteroides	0.97	1.17	0.80	0.878
Erysipelatoclostridium	0.60	1.11	0.45	0.470
Akkermansia	0.72	0.92	0.42	0.756
Dorea	0.82	0.91	0.61	0.930
Phascolarctobacterium	1.36	0.76	0.51	0.465
Lachnoclostridium	0.26	0.41	0.19	0.630
Faecalibacterium	7.24	0.39	2.98	**0.081**
Blautia	2.83	0.26	0.81	0.190
Faecalicoccus	1.14	0.15	0.59	0.663
Clostridium sensu stricto 2	0.01	0.12	0.04	0.354
Subdoligranulum	11.1	0.11	5.09	**0.077**
Fournierella	0.58	0.05	0.26	0.383
[Ruminococcus] torques group	1.42	0.04	0.62	0.507
Pseudoflavonifractor	0.27	0.01	0.01	**0.077**
Alistipes	0.16	0.00	0.05	**0.077**
Others	9.85	9.96	6.57	1.000

Day − 3, 3 days before rotavirus inoculation; Day + 1, 1 day post-inoculation; Day + 7, 7 days post-inoculation.
Healthy: non-diarrhea.
Rota: calves inoculated with rotavirus to induce diarrhea.
SEM: standard error of the mean.
UG: unclassified genus

Table S4. Relative abundance (≥ 0.1%) of fecal bacterial phyla, families, and genera between Healthy and Rota groups post-inoculation day 1

**Measurements**	**Relative abundance (%)**		**SEM**	***p* Value**
	**Healthy**	**Rota**		
Day + 1 Phyla				
Firmicutes	62.2	67.4	13.9	0.823
Verrucomicrobiota	6.11	11.4	7.07	1.000
Proteobacteria	10.2	9.92	7.65	0.663
Bacteroidota	10.3	5.13	4.19	0.437
Fusobacteriota	0.13	4.69	2.38	1.000
Actinobacteriota	10.5	1.23	2.28	**0.064**
Desulfobacterota	0.00	0.23	0.12	1.000
Campylobacterota	0.48	0.00	0.24	1.000
Synergistota	0.07	0.00	0.03	0.505
Day + 1 Families				
Streptococcaceae	10.2	39.9	11.3	0.151
Lactobacillaceae	1.47	20.05	10.5	0.383
Akkermansiaceae	2.67	11.4	6.90	0.825
Enterobacteriaceae	8.50	7.44	6.61	0.663
Fusobacteriaceae	0.13	4.69	2.38	1.000
Bacteroidaceae	7.63	4.56	4.46	0.652
Enterococcaceae	7.38	2.11	2.58	0.256
Clostridiaceae	12.0	1.84	3.94	0.383
Pasteurellaceae	0.62	1.58	1.05	0.663
Lachnospiraceae	3.60	0.33	1.27	0.383
Tannerellaceae	1.36	0.33	0.76	0.643
Acidaminococcaceae	0.22	0.29	0.21	0.839
Peptostreptococcaceae	0.01	0.26	0.11	0.354
Coriobacteriaceae	1.86	0.11	0.83	0.184
Erysipelatoclostridiaceae	0.51	0.10	0.20	0.383
Bifidobacteriaceae	8.53	0.03	2.42	0.190
Ruminococcaceae	7.03	0.00	2.85	0.184
Butyricicoccaceae	5.07	0.00	2.06	**0.077**
Chlamydiaceae	3.43	0.00	1.67	0.197
Prevotellaceae	0.98	0.00	0.34	**0.064**
Others	16.8	4.92	5.60	0.190
Day + 1 Genera				
Streptococcus	10.2	39.9	11.29	0.151
Limosilactobacillus	0.85	11.46	6.04	0.383
Akkermansia	2.67	11.41	6.90	0.825
Ligilactobacillus	0.62	8.59	4.47	0.383

Escherichia-Shigella	8.50	7.44	6.61	0.663
Fusobacterium	0.13	4.69	2.38	1.000
Bacteroides	7.63	4.56	4.46	0.652
Enterococcus	7.38	2.11	2.58	0.256
Clostridium sensu stricto 1	11.86	1.73	3.91	0.383
Gallibacterium	0.62	1.58	1.05	0.663
Parabacteroides	1.36	0.33	0.76	0.643
Phascolarctobacterium	0.22	0.29	0.21	0.839
Peptostreptococcus	0.01	0.26	0.11	0.354
Blautia	0.47	0.19	0.26	1.000
[Ruminococcus] gnavus group	3.06	0.13	1.05	0.184
Clostridium sensu stricto 2	0.10	0.11	0.05	0.663
Collinsella	1.86	0.11	0.83	0.184
Erysipelatoclostridium	0.51	0.10	0.20	0.383
Bifidobacterium	8.53	0.03	2.42	0.190
Ruminococcus	0.21	0.00	0.05	0.184
Butyricicoccus	5.07	0.00	2.06	**0.077**
Chlamydia	3.43	0.00	1.67	0.197
Fournierella	0.86	0.00	0.26	0.197
Faecalibacterium	5.95	0.00	2.80	0.197
Alloprevotella	0.98	0.00	0.34	**0.064**
Others	16.9	4.93	5.59	0.190

Day − 3, 3 days before rotavirus inoculation; Day + 1, 1 day post-inoculation; Day + 7, 7 days post-inoculation.
Healthy: non-diarrhea.
Rota: calves inoculated with rotavirus to induce diarrhea.
SEM: standard error of the mean.

Table S5. Relative abundance (≥ 0.1%) of fecal bacterial phyla, families, and genera between Healthy and Rota groups post-inoculation day 7

Measurements	Relative abundance (%)		SEM	*p* Value
	Healthy	Rota		
Day + 7 Phyla				
Firmicutes	74.29	78.73	6.28	1.000
Actinobacteriota	25.33	12.70	6.45	0.280
Bacteroidota	0.12	3.35	1.56	0.507
Verrucomicrobiota	0.01	2.93	1.47	1.000
Proteobacteria	0.19	2.06	0.87	0.663
Fusobacteriota	0.01	0.21	0.11	1.000
Euryarchaeota	0.04	0.01	0.03	1.000
Day + 7 Families				
Lachnospiraceae	49.1	44.3	18.1	0.862
Streptococcaceae	9.94	14.03	5.83	0.650
Bifidobacteriaceae	23.9	11.8	6.90	0.307
Enterococcaceae	0.43	8.16	2.22	0.081
Clostridiaceae	2.23	4.03	2.73	1.000
Enterobacteriaceae	0.15	1.07	0.45	0.663
Erysipelatoclostridiaceae	3.02	0.43	1.23	0.383
Coriobacteriaceae	0.81	0.37	0.41	1.000
Erysipelotrichaceae	0.11	0.37	0.19	0.825
Eubacteriaceae	0.08	0.17	0.07	0.418
Eggerthellaceae	0.04	0.16	0.07	0.349
Erysipelotrichaceae	0.07	0.09	0.06	0.849
Veillonellaceae	0.11	0.01	0.03	0.354
Others	9.95	15.0	11.1	1.000
Day + 7 Genera				
Limosilactobacillus	37.7	19.9	13.7	0.424
Streptococcus	9.94	14.0	5.83	0.650
Lactobacillus	4.62	12.3	4.98	0.375
Ligilactobacillus	6.46	11.91	5.19	0.508
Bifidobacterium	23.9	11.8	6.90	0.663
Enterococcus	0.43	8.16	2.22	**0.081**
Clostridium sensu stricto 1	2.23	4.03	2.73	1.000
Escherichia-Shigella	0.15	1.07	0.45	0.663
Collinsella	0.81	0.37	0.41	1.000
Turicibacter	0.04	0.28	0.16	1.000
Sharpea	2.95	0.26	1.15	0.190
Eubacterium	0.08	0.17	0.07	0.418
Erysipelatoclostridium	0.07	0.17	0.09	0.469

Paraeggerthella	0.04	0.16	0.07	0.354
Acetitomaculum	0.34	0.10	0.19	0.825
Megasphaera	0.11	0.01	0.03	0.354
Others	10.1	15.2	11.2	1.000

Day − 3, 3 days before rotavirus inoculation; Day + 1, 1 day post-inoculation; Day + 7, 7 days post-inoculation.
Healthy: non-diarrhea.
Rota: calves inoculated with rotavirus to induce diarrhea.
SEM: standard error of the mean.

Table S6. Predicted KEGG hierarchies (orthologs, modules, and pathways) between Healthy and Rota groups

Measurements	Relative abundance (%)		SEM	*p* Value
	Healthy	Rota		
Day − 3				
KEGG orthologs	4987	4647	181	0.274
KEGG pathways	127	126	8.21	0.191
KEGG modules	243	250	5.49	0.258
Day + 1				
KEGG orthologs	4897	5065	253	0.713
KEGG pathways	136	139	7.01	0.783
KEGG modules	140	126	5.44	0.497
Day + 7				
KEGG orthologs	4633	4371	525	0.759
KEGG pathways	246	234	6.26	0.957
KEGG modules	232	228	13.6	0.874

Day − 3, 3 days before rotavirus inoculation; Day + 1, 1 day post-inoculation; Day + 7, 7 days post-inoculation.
Healthy: non-diarrhea.
Rota: calves inoculated with rotavirus to induce diarrhea.
SEM: standard error of the mean.
KEGG: Kyoto Encyclopedia of Genes and Genomes.

Table S7. Differentially abundant KEGG modules between Healthy and Rota groups, which were detected using LEfSe with an LDA effect size > 2.

KEGG modules	Dominance	Treatment		SEM	LDA	*p* Value	Description
		Healthy	Rota				
Day − 3							
M00176	Healthy	0.24	0.12	0.03	3.003	0.050	Assimilatory sulfate reduction, sulfate => H2S
M00922	Healthy	0.25	0.02	0.06	3.093	0.050	CMP-Neu5Ac biosynthesis
M00923	Healthy	0.14	0.01	0.03	2.954	0.050	UDP-L-FucNAm biosynthesis
M00895	Rota	0.73	0.82	0.02	2.881	0.050	Thiamine biosynthesis, prokaryotes, AIR (+ DXP/glycine) => TMP/TPP
M00899	Rota	0.74	0.92	0.03	3.095	0.050	Thiamine salvage pathway, HMP/HET => TMP
Day + 1							
M00061	Healthy	0.56	0.17	0.08	3.337	0.050	D-Glucuronate degradation, D-glucuronate => pyruvate + D-glyceraldehyde 3P
M00133	Healthy	0.34	0.10	0.03	3.223	0.050	Polyamine biosynthesis, arginine => agmatine => putrescine => spermidine
M00135	Healthy	0.26	0.05	0.06	3.098	0.050	GABA biosynthesis, eukaryotes, putrescine => GABA
M00308	Healthy	0.94	0.74	0.04	3.092	0.050	Semi-phosphorylative Entner-Doudoroff pathway, gluconate => glycerate-3P
M00345	Healthy	0.83	0.58	0.07	3.208	0.050	Formaldehyde assimilation, ribulose monophosphate pathway
M00631	Healthy	0.54	0.17	0.08	3.300	0.050	D-Galacturonate degradation (bacteria), D-galacturonate => pyruvate + D-glyceraldehyde 3P
M00651	Healthy	0.20	0.14	0.01	2.792	0.050	Vancomycin resistance, D-Ala-D-Lac type
M00845	Healthy	0.81	0.71	0.04	3.063	0.050	Arginine biosynthesis, glutamate => acetylcitrulline => arginine
M00878	Healthy	0.16	0.09	0.02	2.859	0.050	Phenylacetate degradation, phenylaxetate => acetyl-CoA/succinyl-CoA
M00892	Healthy	0.91	0.53	0.14	3.315	0.050	UDP-N-acetyl-D-glucosamine biosynthesis, eukaryotes, glucose => UDP-GlcNAc
M00032	Rota	0.15	0.19	0.01	3.022	0.050	Lysine degradation, lysine => saccharopine => acetoacetyl-CoA
M00126	Rota	0.61	0.75	0.03	3.053	0.050	Tetrahydrofolate biosynthesis, GTP => THF
M00129	Rota	0.34	0.47	0.04	3.000	0.050	Ascorbate biosynthesis, animals, glucose-1P => ascorbate
M00150	Rota	0.23	0.62	0.09	3.363	0.050	Fumarate reductase, prokaryotes
M00168	Rota	0.33	0.63	0.11	3.219	0.050	CAM (Crassulacean acid metabolism), dark
M00346	Rota	0.66	0.74	0.03	2.849	0.050	Formaldehyde assimilation, serine pathway

M00793	Rota	0.93	1.25	0.09	3.174	0.050	dTDP-L-rhamnose biosynthesis
M00840	Rota	0.65	0.91	0.05	3.277	0.050	Tetrahydrofolate biosynthesis, mediated by ribA and trpF, GTP => THF
M00842	Rota	0.59	0.84	0.04	3.186	0.050	Tetrahydrobiopterin biosynthesis, GTP => BH4
M00843	Rota	0.59	0.84	0.04	3.186	0.050	L-threo-Tetrahydrobiopterin biosynthesis, GTP => L-threo-BH4
M00899	Rota	0.75	0.99	0.05	3.225	0.050	Thiamine salvage pathway, HMP/HET => TMP
M00913	Rota	0.55	0.79	0.07	3.201	0.050	Pantothenate biosynthesis, 2-oxoisovalerate/spermine => pantothenate
Day + 7							
M00017	Healthy	1.32	1.06	0.05	3.077	0.050	Methionine biosynthesis, aspartate => homoserine => methionine
M00018	Healthy	1.25	1.08	0.04	2.792	0.050	Threonine biosynthesis, aspartate => homoserine => threonine
M00029	Healthy	1.13	0.97	0.05	3.072	0.050	Urea cycle
M00526	Healthy	1.39	1.14	0.06	2.914	0.050	Lysine biosynthesis, DAP dehydrogenase pathway, aspartate => lysine
M00527	Healthy	1.39	1.14	0.06	3.026	0.050	Lysine biosynthesis, DAP aminotransferase pathway, aspartate => lysine
M00046	Rota	0.08	0.13	0.01	3.073	0.050	Pyrimidine degradation, uracil => beta-alanine, thymine => 3-aminoisobutanoate
M00117	Rota	0.92	1.03	0.04	2.817	0.050	Ubiquinone biosynthesis, prokaryotes, chorismate (+ polyprenyl-PP) => ubiquinol
M00373	Rota	0.46	0.62	0.04	2.603	0.050	Ethylmalonyl pathway
M00530	Rota	0.05	0.25	0.05	3.083	0.050	Dissimilatory nitrate reduction, nitrate => ammonia
M00880	Rota	0.05	0.18	0.02	3.031	0.050	Molybdenum cofactor biosynthesis, GTP => molybdenum cofactor
M00899	Rota	0.02	0.22	0.04	3.011	0.050	Thiamine salvage pathway, HMP/HET => TMP
M00913	Rota	0.10	0.28	0.04	2.873	0.050	Pantothenate biosynthesis, 2-oxoisovalerate/spermine => pantothenate

Day − 3, 3 days before rotavirus inoculation; Day + 1, 1 day post-inoculation; Day + 7, 7 days post-inoculation.
Healthy: non-diarrhea.
Rota: calves inoculated with rotavirus to induce diarrhea.
SEM: standard error of the mean.
KEGG: Kyoto Encyclopedia of Genes and Genomes; LDA: linear discriminant analysis; LEfSe: LDA effect size.
